# Supplementary material for: A High-Copy Suppressor Screen Reveals a Broad Role of Prefoldin-like Bud27 in the TOR Signaling Pathway in Saccharomyces cerevisiae
Source: Genes (Basel). 2022 Apr 24;13(5):748. doi: 10.3390/genes13050748 (PMC9141189; doi:10.3390/genes13050748)
Supplement: Supplementary file 1 [file genes-13-00748-s001.zip › Figures S1.pdf]

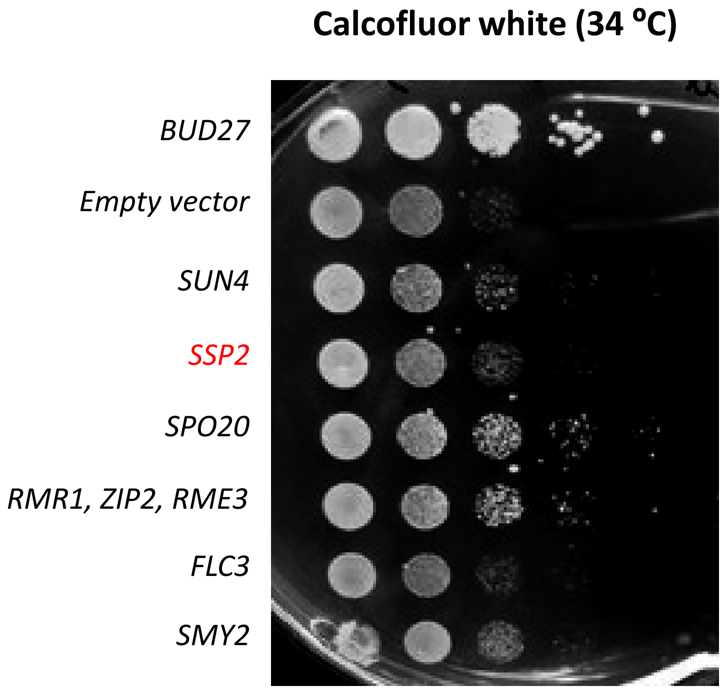

Figure S1. Some suppressors overcome the sensitivity of the *bud27Δ* mutant to calcofluor. A) Growth of the *bud27Δ* mutant at 34ºC in SD minimal medium with calcofluor white (10 mg/ml), harboring a multicopy empty vector or multicopy vectors allowing overexpression of the indicated genes. Red denotes a putative suppressor gene.
