# Supplementary material for: A High-Copy Suppressor Screen Reveals a Broad Role of Prefoldin-like Bud27 in the TOR Signaling Pathway in Saccharomyces cerevisiae
Source: Genes (Basel). 2022 Apr 24;13(5):748. doi: 10.3390/genes13050748 (PMC9141189; doi:10.3390/genes13050748)
Supplement: Supplementary file 1 [file genes-13-00748-s001.zip › Table Sup (STRING).pdf]

Supplemental Table S1

Functional categories (Biological Process) for the up-regulated genes in *bud27Δ* mutant vs wild-type strain (≥ 2), and differentially expressed than in a wild-type strain under rapamycin addition [1] .The analysis was performed using the STRING software [2] and the PPI enrichment p-value:<1.0e-16 was selected.

Functional categories	FDR	Functional categories	FDR
Cellular amino acid biosynthetic process	2.37e-13	Aromatic amino acid family metabolic process	4.19e-06
Alpha-amino acid biosynthetic process	4.67e-12	Cellular biosynthetic process	4.19e-06
Small molecule metabolic process	8.92e-12	Cellular metabolic process	2.87e-05
Small molecule biosynthetic process	8.92e-12	Lysine biosynthetic process via aminoadipic acid	3.88e-05
Carboxylic acid biosynthetic process	8.92e-12	Organic cyclic compound biosynthetic process	5.50e-05
Oxoacid metabolic process	3.01e-11	Metabolic process	7.74e-05
Cellular amino acid metabolic process	3.18e-11	Organic substance metabolic process	0.00010
Cellular process	3.43e-11	Aromatic compound biosynthetic process	0.00033
Alpha-amino acid metabolic process	3.97e-11	Sulfur amino acid metabolic process	0.0027
Carboxylic acid metabolic process	1.46e-10	Organonitrogen compound biosynthetic process	0.0041
Aspartate family amino acid metabolic process	1.73e-07	Heterocycle biosynthetic process	0.0071
Aspartate family amino acid biosynthetic process	8.48e-07	Methionine metabolic process	0.0072
Organic substance biosynthetic process	1.70e-06	Sulfur compound biosynthetic process	0.0097
Lysine biosynthetic process	2.08e-06		
Aromatic amino acid family biosynthetic process	2.23e-06		

Supplemental Table S2

Functional categories (Biological Process) for the down-regulated genes in *bud27Δ* mutant vs wild-type strain (≥ 2), and differentially expressed than in a wild-type strain under rapamycin addition [1] .The analysis was performed using the STRING software [2] and the PPI enrichment p-value:<1.0e-16 was selected.

Functional categories	FDR
Fungal-type cell wall organization	2.50e-05
Cell wall organization	2.50e-05
Cell wall organization or biogenesis	4.04e-05
Fungal-type cell wall organization or biogenesis	4.04e-05
Carbohydrate derivative metabolic process	0.0018
Transmembrane transport	0.0032
Cell wall biogenesis	0.0089

1. Cuevas-Bermúdez, A.; Garrido-Godino, A.; Navarro, F., A novel yeast chromatin-enriched fractions purification approach, yChEFs, for the chromatin-associated protein analysis used for chromatin-associated and RNA-dependent chromatin-associated proteome studies from *Saccharomyces cerevisiae*. *Gene Reports* **2019,** 16, 100450.
2. Szklarczyk, D.; Morris, J. H.; Cook, H.; Kuhn, M.; Wyder, S.; Simonovic, M.; Santos, A.; Doncheva, N. T.; Roth, A.; Bork, P.; Jensen, L. J.; von Mering, C., The STRING database in 2017: quality-controlled protein-protein association networks, made broadly accessible. *Nucleic Acids Res* **2017,** 45, (D1), D362-D368.
